# Reirradiation of head and neck cancer: Long‐term disease control and toxicity

**DOI:** 10.1002/hed.24733

**Published:** 2017-03-06

**Authors:** Wouter T. C. Bots, Sven van den Bosch, Ellen M. Zwijnenburg, Tim Dijkema, Guido B. van den Broek, Willem L. J. Weijs, Lia C. G. Verhoef, Johannes H. A. M. Kaanders

**Affiliations:** ^1^ Department of Radiation OncologyRadboud University Medical CenterNijmegenThe Netherlands; ^2^ Department of Otorhinolaryngology Head and Neck SurgeryRadboud University Medical CenterNijmegenThe Netherlands; ^3^ Department of Oral and Maxillofacial SurgeryRadboud University Medical CenterNijmegenThe Netherlands

**Keywords:** reirradiation, head and neck cancer, intensity‐modulated radiotherapy (IMRT), late toxicity, disease control

## Abstract

**Background:**

The purpose of this study was to report long‐term disease control and late radiation toxicity for patients reirradiated for head and neck cancer.

**Methods:**

We conducted a retrospective analysis of 137 patients reirradiated with a prescribed dose ≥45 Gy between 1986 and 2013 for a recurrent or second primary malignancy. Endpoints were locoregional control, overall survival (OS), and grade ≥4 late complications according to European Organization for Research and Treatment of Cancer (EORTC)/Radiation Therapy Oncology Group (RTOG) criteria.

**Results:**

Five‐year locoregional control rates were 46% for patients reirradiated postoperatively versus 20% for patients who underwent reirradiation as the primary treatment (*p* < .05). Sixteen cases of serious (grade ≥4) late toxicity were seen in 11 patients (actuarial 28% at 5 years). In patients reirradiated with intensity‐modulated radiotherapy (IMRT), a borderline improved locoregional control was observed (49% vs 36%; *p* = .07), whereas late complication rates did not differ.

**Conclusion:**

Reirradiation should be considered for patients with a recurrent or second primary head and neck cancer, especially postoperatively, if indicated. © 2017 Wiley Periodicals, Inc. *Head Neck* **39:** 1122–1130, 2017

## INTRODUCTION

Up to 40% of patients treated for head and neck cancer develop a recurrence within 5 years after treatment.^1^, ^2^ In addition, the probability of developing a second primary tumor in the head and neck area is approximately 20%, frequently associated with a history of tobacco and/or alcohol abuse.^3^

Traditionally, surgery is the treatment of choice for locoregional recurrences of head and neck cancer in a previously irradiated area.^4^, ^5^ However, surgery is not always a feasible option because of irresectability of the tumor in advanced stages or the condition of the patient not allowing surgery. Therefore, reirradiation often is the only possible alternative with curative intent. Furthermore, even after surgery, reirradiation may still be indicated in patients with adverse histopathologic features, such as positive resection margins or nodal metastases with extracapsular extension.^6^

Over the last decade, reirradiation has gained more acceptance. As a result, patients who currently develop a recurrence or a second primary malignancy are increasingly being considered for reirradiation. An important reason for this trend is that highly conformal irradiation techniques, such as intensity‐modulated radiotherapy (IMRT)/volumetric‐modulated arc therapy (VMAT), allow better sparing of uninvolved tissue.^7^

A major drawback of reirradiation in the head and neck region remains the concern for severe late radiation toxicity. This includes extensive fibrosis, soft tissue necrosis, osteoradionecrosis (ORN), myelopathy, and carotid artery blowout. In literature, serious (European Organization for Research and Treatment of Cancer [EORTC]/Radiation Therapy Oncology Group [RTOG] grade 3 or higher) late treatment complication rates of up to 50% are reported, although rates vary greatly because of heterogeneous study populations.^8^, ^9^, ^10^, ^11^, ^12^

This retrospective single center study includes one of the largest cohorts of patients reirradiated for head and neck tumors with a long‐term follow‐up. The purpose of this study was to gain more insight on disease control and late radiation toxicity in both primary and postoperative reirradiation in the head and neck region. This will help to determine which patients will benefit the most from reirradiation and if IMRT indeed reduces the risk of severe late toxicity.

## MATERIALS AND METHODS

### Patient selection

The medical records of 167 consecutive patients who were reirradiated to the head and neck region between 1986 and 2013 for a recurrent or second primary malignancy were analyzed. All patients were treated at the Radboud University Medical Center, Nijmegen, The Netherlands. The date of last data collection was June 2015. Inclusion criteria were external‐beam radiotherapy with a prescribed dose of at least 45 Gy in both primary treatment and re‐treatment, and histological proof of disease before both treatments.

Exclusion criteria were age <18 years at re‐treatment, brachytherapy as part of one or both treatments, or the presence of metastatic disease. A total of 30 patients were excluded because of missing data (*n* = 12) or absence of overlap of radiation volumes (*n* = 18), leaving 137 evaluable patients (Table 1).

**Table 1:** Patient and tumor characteristics for the entire cohort and for subgroups of patients receiving reirradiation as postoperative treatment or reirradiation alone.

			Subgroups			
Variables	Total (*n* = 137)		Postoperative RT (*n* = 108)		Primary RT (*n* = 29)	
Characteristics						
Male patients	113	82%	88	81%	25	86%
Age, y (range)	65	(31–88)	65	(31–88)	64	(47–83)
Median follow‐up, mo (95% CI)	46	(33–59)	46	(31–61)	59	(15–103)
Median time in mo between RT and re‐RT (range)	23	(6–296)	21	(6–296)	31	(13–179)
Median dose first RT in Gy (range)	68	(45–74)	68	(45–74)	68	(60–70)
Median dose re‐RT in Gy (range)	60	(45–70)	60	(45–70)	60	(48–70)
Median cumulative dose in Gy (range)	126	(70–138)	126	(70–136)	124	(76–138)
Chemotherapy at re‐RT	7	5%	7	6%	0	0%
Second primary tumor	45	33%	31	29%	14	48%
Use of IMRT for re‐RT	60	44%	53	49%	7	24%
Elective neck radiated at first RT	88	64%	71	66%	17	59%
Initial tumor T classification						
0	1	1%	1	1%	0	0%
1	33	24%	26	24%	7	24%
2	60	44%	45	42%	15	52%
3	23	17%	19	18%	4	14%
4	20	15%	17	16%	3	10%
Initial tumor N classification						
0	88	64%	66	61%	22	76%
1	16	12%	14	13%	2	7%
2	32	23%	28	26%	4	14%
3	1	1%	0	0%	1	3%
Initial tumor site						
Larynx	73	53%	55	51%	18	62%
Oral cavity	19	14%	16	15%	3	10%
Oropharynx	17	12%	13	12%	4	14%
Hypopharynx	9	7%	8	7%	1	3%
Paranasal sinus	7	5%	6	6%	1	3%
Nasal vestibule	5	4%	4	4%	1	3%
Nasopharynx	3	2%	3	3%	0	0%
Other	4	3%	3	3%	1	3%
Histology						
Squamous cell carcinoma	118	86%	91	84%	27	93%
Mucoepidermoid carcinoma	4	3%	4	4%	0	0%
Adenocarcinoma	3	2%	3	3%	0	0%
Other	12	9%	10	9%	2	7%
Recurrent T classification						
0	44	32%	39	36%	5	17%
1	12	9%	8	7%	4	14%
2	23	17%	16	15%	7	24%
3	15	11%	11	10%	4	14%
4	43	31%	34	32%	9	31%
Recurrent N classification						
0	70	51%	50	46%	20	69%
1	35	26%	32	30%	3	10%
2	32	23%	26	24%	6	21%
3	0	0%	0	0%	0	0%
Surgery before re‐RT						
Laryngectomy			44	41%		
Selective neck dissection			68	63%		
Local resection			28	26%		
Postoperative histology						
Close/positive margins			74	69%		
Extracapsular extension			38	35%		
Perineural invasion			21	19%		
Re‐RT tumor site						
Larynx	40	29%	30	28%	10	35%
Oral cavity	11	8%	8	7%	3	10%
Oropharynx	10	7%	9	8%	1	3%
Hypopharynx	10	7%	8	7%	2	7%
Paranasal sinus	3	2%	2	2%	1	3%
Nasal vestibule	4	3%	4	4%	0	0%
Nasopharynx	5	4%	4	4%	1	3%
Neck	49	36%	42	39%	7	24%
Other	5	4%	1	1%	4	14%
Tumor recurrence type						
Local only	70	51%	50	46%	20	69%
Nodal (single node)	28	20%	27	25%	1	3%
Nodal (multiple nodes)	16	12%	12	11%	4	14%
Both local and nodal	23	17%	19	18%	4	14%

Abbreviations: RT, radiotherapy; 95% CI, 95% confidence interval; re‐RT, re‐radiotherapy; IMRT, intensity‐modulated radiotherapy.

Before treatment, all patients were evaluated by physical examination and radiologic imaging (CT or MRI and ultrasound of the neck). Screening for distant metastasis was performed primarily by chest X‐ray. In case of high nodal classification and/or lymph node metastasis in the lower neck levels, a chest CT or positron emission tomography‐CT was performed. All patients were discussed in a multidisciplinary consensus conference for staging and treatment recommendations. The board was comprised of head and neck surgeons, radiation oncologists, medical oncologists, radiologists, pathologists, and a nuclear medicine physician. For reirradiation, several patient factors were taken into account, such as comorbidity, toxicity of previous radiation, and the time interval since the previous treatment. The minimum interval had to be 6 months, although before the year 2010 a 1‐year interval was generally preferred. A recurring tumor was defined as a second primary if it was located at least 2 cm from the index primary cancer or if it occurred more than 5 years after the index primary. During the latter part of the study period, *TP53* mutation analysis was increasingly used to discriminate a recurrence from a second primary tumor.

### Treatment guidelines

Patients were treated with conventional 2D radiotherapy, a 3D conformal technique, or IMRT, according to standard practice of that time. From 2005 onward, IMRT was gradually introduced, and was used for 31 patients (23%) during the first treatment and for 60 patients (44%) during the reirradiation. IMRT was used for both the primary and reirradiation treatments in 30 patients.

During the treatments, patients were immobilized with a thermoplastic head, neck, and shoulder mask. A 5 to 15 mm expansion (depending on the pattern of spread and adjusted for natural anatomic borders) around the gross tumor volume defined the clinical target volume (CTV). In postoperative settings, the CTV contained the entire surgical bed. Neck nodes were electively irradiated, and the levels included were dependent on the site and extensions of the primary tumor and nodal classification. The planning target volume was created by extension of the CTV with a margin of 3 to 5 mm. The most common indications for postoperative reirradiation were close (<5 mm but ≥1 mm) or positive (<1 mm) resection margins or extracapsular extension of lymph node metastasis.

For the first radiation treatment, patients were treated strictly by protocol. Up to 1996, dose prescribed to gross tumor sites was 68 to 70 Gy in 2 Gy fractions and elective dose was 44 Gy in 2 Gy fractions. As per 1996, accelerated fractionation was introduced reducing overall treatment time from 7 weeks to 5.5 weeks. With the gradual introduction of IMRT from 2005 onward, the elective dose was adjusted to 50 Gy in 1.47 Gy fractions because an integrated boost technique was introduced. Patients irradiated postoperatively received a dose of 64 to 66 Gy in 2 Gy fractions to high‐risk areas and 50 to 54 Gy to intermediate‐risk areas using a conventional fractionation schedule.

With primary reirradiation, dose to gross tumor was typically 60 to 66 Gy and 50 Gy to the elective neck, which was treated in the majority of the cases. Postoperative reirradiation dose to high‐risk areas was 60 Gy. However, reirradiation target volumes and prescribed doses were often individualized depending on normal tissue constraints, previous given dose, interval between treatments, and clinical signs of radiation‐induced damage to previously irradiated tissues.

Cumulative dose constraints of 60 Gy (equivalent 2 Gy dose) were applied to the spinal cord and brain stem. A 50% dose tolerance recovery was assumed after a radiotherapy interval of at least 12 months.^13^ Concurrent chemotherapy was administered in 7 patients (5%) during reirradiation (Table 1). Regimens contained cisplatin, carboplatin, or 5‐fluorouracil.

### Dose registration

Reirradiation was defined as the overlap between initial and re‐treatment target volumes. To determine the cumulative radiation dose in the overlapping volumes, a descriptive approach was used. This consisted of visually comparing treatment plans of both the first and second treatment, and carefully estimating the area with the highest summed radiation dose. For this area, the cumulative maximum physical radiation dose was reported. If the overlapping areas did not receive the full prescribed dose (eg, electively treated areas), the summed dose was lower than the mathematical sum of the prescribed doses for each treatment. For digitally stored plans, Pinnacle version 9.10 (Philips Radiation Oncology Systems, Fitchburg, WI) was used. For 2D techniques, analog simulation films were used. Because of the incompleteness of volume data for patients treated in the earlier years, we could not analyze dose‐volume relationships. Organs at risk specifically assessed were the spinal cord, larynx, and mandible. The maximum summed radiation point dose in any organ at risk over the 2 treatments was estimated.

### Survival and toxicity endpoints

Medical files were retrieved and analyzed for disease recurrence, treatment complications, and cause of death if available. For patients lost to follow‐up, the Dutch population registry was enquired to retrieve survival status and date of death, if applicable.

Regular oncologic follow‐up visits were planned every 2, 3, 4, 5, and 6 months during the first, second, third, fourth, and fifth years after treatment, respectively. The tumor recurrence date was defined as the date of histological confirmation of the recurrence.

Late toxicity was scored according to the EORTC/RTOG late radiation toxicity criteria^14^ for the following tissues: skin, subcutaneous tissue, mucous membrane, spinal cord, brain, eyes, larynx, esophagus, and bones. Only the severe radiation‐related toxicities, defined as grade 4 or higher, and occurring or persisting after 6 months were considered.

### Statistical analysis

Overall survival (OS), locoregional control, disease‐free survival (DFS), event‐free survival, and late complication rates were calculated from the first day of reirradiation, according to the method of Kaplan–Meier. Median follow‐up for surviving patients is reported using the reverse Kaplan–Meier method.^15^ The log‐rank test was used to compare subgroups. In the situation of multiple late complications in 1 patient, the date of the first occurring complication was regarded as an event. In order to identify the proportion of patients without disease recurrence or late complications, the event‐free survival was calculated. Events for event‐free survival were both disease recurrence and treatment complications. The Mann–Whitney *U* test was used to compare skewed data. Univariate analyses using the Cox regression model were performed to determine predictors of locoregional control, OS, and late complications for the following subgroups: age at reirradiation <65 versus ≥65 years; laryngeal tumor location versus other; primary versus postoperative reirradiation; recurrence versus second primary malignancy; reirradiation dose <60 versus ≥60 Gy; given cumulative radiation dose <126 versus ≥126 Gy; IMRT versus conventional reirradiation technique; concurrent chemotherapy versus not with reirradiation; interval between treatments <3 versus ≥3 years; and recurrent N0 to N1 category versus N2 to N3 category. Multivariate analysis for the subgroups was performed for *p* values < .10 in univariate analysis using the Cox regression model for the endpoints locoregional control and OS. Using the Mann–Whitney *U* test, a *p* value of < .05 was considered to be statistically significant. Statistical analysis was performed in the SPSS version 22.0 software program (IBM SPSS Statistics for Windows, Armonk, NY).

## RESULTS

**Figure 1:**
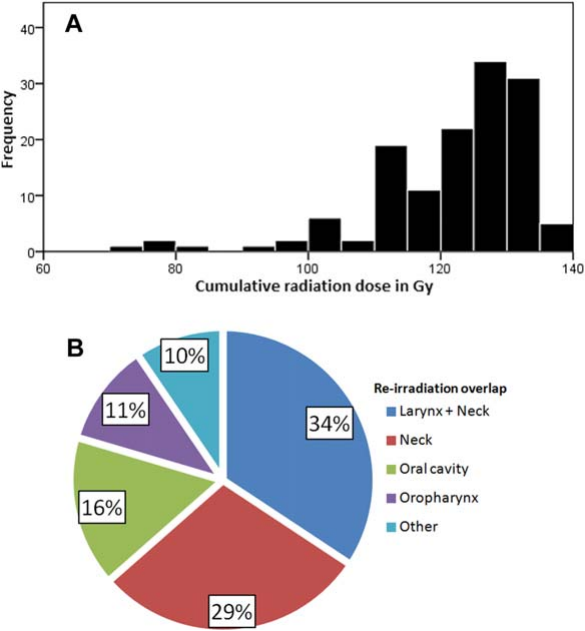
**(A) Distribution of cumulative re‐irradiation dose (*n* = 137). (B) Distribution of reirradiation overlap areas.**

Patient characteristics are given in Table 1. Distribution of cumulative radiation doses and overlap of radiated areas are visualized in Figure 1. The median duration of follow‐up of surviving patients was 46 months. Median follow‐up of surviving patients reirradiated with IMRT was 34 months compared with 77 months with conventional techniques.

### Overall survival

**Figure 2:**
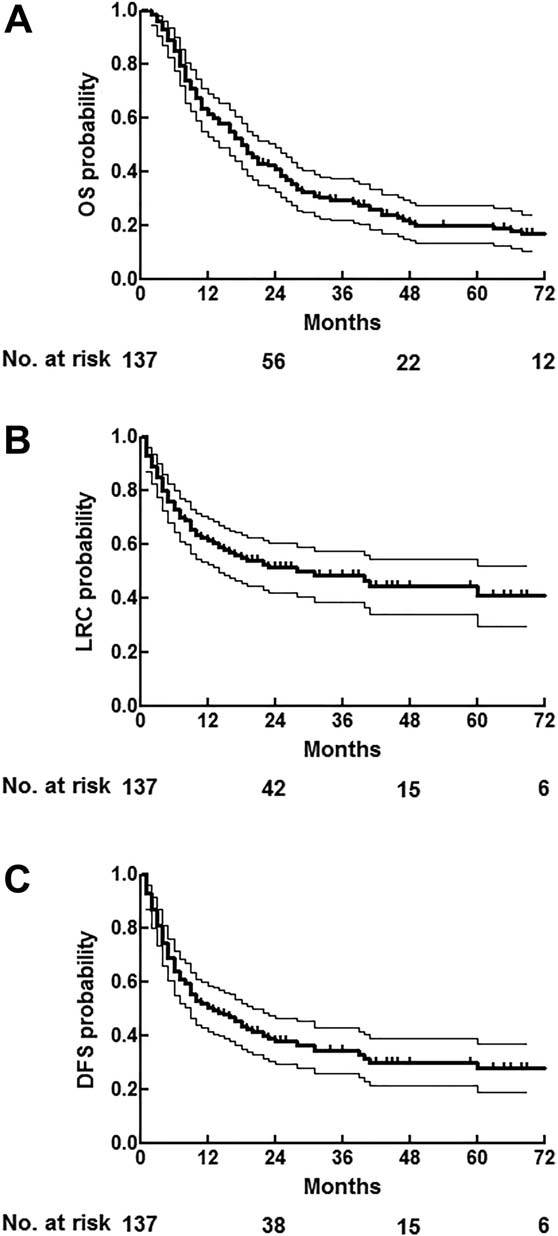
**(A) Overall survival with 95% confidence interval (CI). (B) Overall locoregional control with 95% CI. (C) Overall disease‐free survival with 95% CI.**

**Figure 3:**
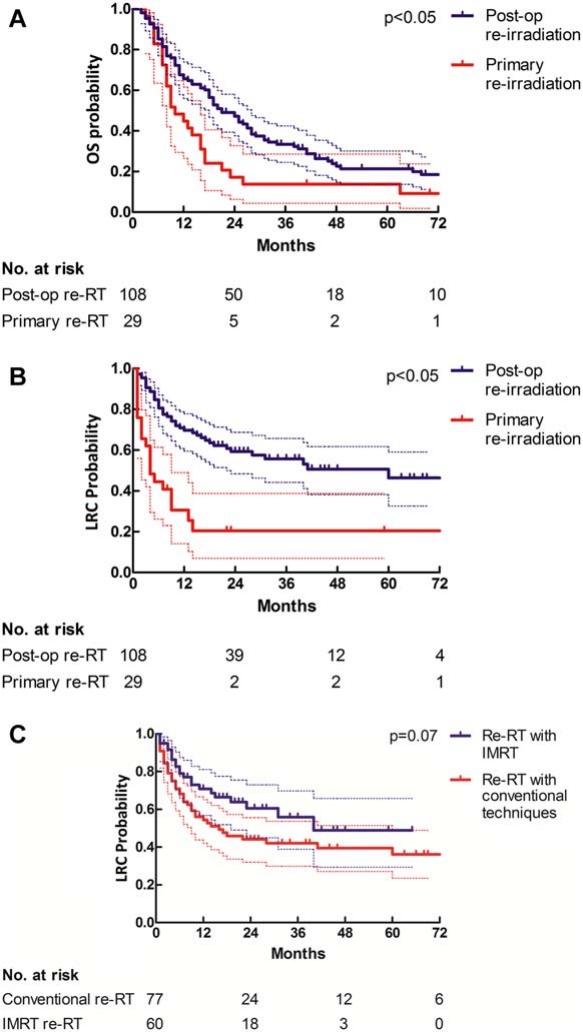
**(A) Overall survival in primary tumor and postoperative (post‐op) reirradiation (re‐RT) with 95% confidence interval (CI). (B) Locoregional control in primary tumor and postoperative reirradiation with 95% CI. (C) Locoregional control in patients reirradiated with intensity‐modulated radiotherapy (IMRT) versus conventional techniques with 95% CI.**

For the entire cohort, 2‐year and 5‐year actuarial OS rates were 42% and 20%, respectively (Figure 2A). Patients who underwent reirradiation alone had a worse 2‐year OS (17% 2‐year; median OS, 10 months; 95% confidence interval [CI], 4–15 months) in comparison with patients reirradiated postoperatively (48% 2‐year; median OS, 21 months; 95% CI, 15–26 months). The difference in OS at 5 years was not as prominent but still significant (14% vs 21% 5‐year; *p* = .01; Figure 3A). Five‐year OS rates were similar for patients reirradiated with IMRT (19%) in comparison with conventional techniques (19%; *p* = .32). In multivariate analysis, the variables were: patient age 65 years or lower; postoperative reirradiation; radiation interval of more than 3 years; or a recurrent N classification of 0 to 1 versus 2 to 3 were associated with a higher OS (Table 2).

**Table 2:** Locoregional control and overall survival univariate and multivariate analyses.

		Univariate analysis			Multivariate analysis		
Variables	Code	Hazard ratio	95% CI	*p* value	Hazard ratio	95% CI	*p* value
OS							
Age at reirradiation	≥ vs <65 y	1.46	0.99–2.13	.05^a^	1.49	1.01–2.18	.04^a^
Tumor location	Laryngeal vs other	0.99	0.91–1.10	.93			
Reirradiation	Postop vs primary	0.56	0.36–0.88	.01^a^	0.46	0.29–0.74	.001^a^
Recurrence	Second primary vs recurrence	0.73	0.48–1.10	.13			
Reirradiation dose	≥60 vs <60 Gy	1.13	0.77–1.65	.54			
Cumulative radiation dose	≥126 vs <126 Gy	1.15	0.79–1.67	.47			
Reirradiation technique	IMRT vs conventional	0.82	0.56–1.21	.32			
Concurrent chemotherapy	Yes vs none	0.59	0.22–1.56	.30			
Interval between treatments	≥3 vs <3 y	0.62	0.41–0.93	.02^a^	0.56	0.37–0.87	.01^a^
Recurrent N classification	T×N2–N3 vs T×N0–N1	1.67	1.09–2.58	.02^a^	1.53	0.99–2.36	.06
Locoregional control							
Age at reirradiation	≥ vs <65 y	0.81	0.49–1.33	.40			
Tumor location	Laryngeal vs other	0.84	0.51–1.37	.47			
Reirradiation	Postop vs primary	0.30	0.17–0.51	.0001^a^	0.31	0.18–0.53	.0001^a^
Recurrence	Second primary vs recurrence	0.82	0.48–1.40	.47			
Reirradiation dose	≥60 vs < 60 Gy	1.00	0.61–1.64	.99			
Cumulative radiation dose	≥126 vs < 126 Gy	0.85	0.52–1.40	.53			
Reirradiation technique	IMRT vs conventional	0.62	0.37–1.04	.07	0.65	0.39–1.10	.11
Concurrent chemotherapy	Yes vs none	0.82	0.26–2.60	.73			
Interval between treatments	≥3 vs < 3 y	0.84	0.50–1.42	.52			
Recurrent N classification	T×N2–3 vs T×N0–1	1.67	0.96–2.89	.07	1.72	0.99–2.99	.06

Abbreviations: 95% CI, 95% confidence interval; OS, overall survival; Postop, postoperative; IMRT, intensity‐modulated radiotherapy.

a *p* < .05.

### Locoregional control

Actuarial 2‐year and 5‐year locoregional control rates were 51% and 41%, respectively, for the entire cohort (Figure 2B). As shown in Figure 3B, patients reirradiated without prior surgery had a worse 2‐year and 5‐year locoregional control rates (20% 2‐year; 20% 5‐year) in comparison with patients reirradiated postoperatively (59% 2‐year; 46% 5‐year; *p* < .05; Table 2). Of the 29 patients primarily reirradiated without prior surgical intervention, 14 patients were reirradiated for a second primary tumor. In this group, the 5‐year locoregional control rate was favorable at 44%. In contrast, the remaining 15 patients who were primarily reirradiated for a recurring tumor either had a locoregional recurrence within 2 years after treatment (*n* = 14) or died of an unknown cause (*n* = 1). The most commonly performed surgery before reirradiation was laryngectomy in 44 patients. In this group of patients, the 2‐year and 5‐year locoregional control rates were 69% and 59%, respectively.

The 2‐year locoregional control rate of patients undergoing reirradiation after an isolated nodal recurrence (27/28 patients treated postoperatively) was 54%. The 2‐year locoregional control rates for patients with an isolated local recurrence (rT+N0), nodal recurrence without evidence of local disease (rT0N+), or both (rT+N+) were not significantly different (53% vs 49% vs 49%; *p* = .98).

### Intensity‐modulated radiotherapy

For the whole cohort, univariate analysis revealed borderline improved long‐term locoregional control rates in patients reirradiated with IMRT (49% vs 36%; *p* = .07; Figure 3C), although at multivariate analysis this effect could not be confirmed (Table 2). Median time to locoregional failure was 40 months for patients reirradiated with IMRT versus 16 months for patients treated with conventional techniques. Patients treated with IMRT received a higher median reirradiation (60 Gy vs 56 Gy; *p* < .05) and cumulative radiation dose (128 Gy vs 120 Gy; *p* < .05) in comparison with patients treated with conventional techniques.

Univariate and multivariate analyses revealed no other significant factors associated with locoregional control (Table 2). Univariate analysis of the subgroup of postoperative patients revealed no significant prognostic variables.

### Disease‐free survival

For the entire cohort, the 2‐year and 5‐year actuarial DFS rates were 39% and 27%, respectively (Figure 2C). Postoperatively reirradiated patient had a better 5‐year DFS rates (30%) in comparison with patients reirradiated alone (20%). Thirty patients developed distant metastases, of whom the majority (90%) was diagnosed within 2 years from reirradiation (median, 7 months; range, 2–39 months). Of these 30 patients, 7 developed metastatic disease without evidence of locoregional recurrence, which means that approximately one fourth of these disease recurrences were distant metastases alone.

### Late toxicity

Late complication frequencies are listed in Table 3. In total, 11 patients incurred 16 late complications grade ≥4. At these complication sites, the median cumulative radiation dose was 114 Gy (range, 94–130 Gy). The 5‐year actuarial serious late complication‐free rate was 72% (95% CI, 52% to 92%). Three patients died of a late treatment complication, 2 from an arterial blowout and 1 because of bleeding from the necrotic tissue (cumulative dose, 128–130 Gy; Table 3).

**Table 3:** Grade ≥4 late toxicity rates.

Toxicity grade	IV (major)	V (death)
Osteonecrosis	8	
Chondronecrosis (laryngeal)	1	
Mucosal/subcutaneous tissue	2	1
Arterial blowout	1	2
Fistula	1	

Total: 16

There was no difference in the late toxicity rates between patients receiving postoperative reirradiation and those receiving reirradiation alone (28% vs 24% at 5 years; *p* = .09). The most commonly performed surgery before reirradiation was laryngectomy in 45 patients. In the total cohort, 2 patients required a feeding tube 6 months after reirradiation, 1 of these patients had a laryngectomy with postoperative reirradiation.

Toxicity rates did not significantly differ between patients reirradiated with IMRT or not (5‐year 32%; 95% CI, 9% to 55% vs 12%; 95% CI, 0% to 25%; *p* = .90).

Eight patients developed ORN after reirradiation, and 1 patient developed chondronecrosis of the larynx. The median cumulative radiation dose at these complication sites was 114 Gy (range, 94–130 Gy). Affected sites were the mandible (*n* = 5), clavicle (*n* = 1), base of skull (*n* = 1), cervical vertebra (*n* = 1), and larynx (*n* = 1). The time interval from reirradiation to diagnosis of ORN ranged from 2 to 45 months. In the 5 patients who developed mandibular ORN, the dose range was 104 to 128 Gy. Fifty‐four patients received a cumulative dose of 100 Gy or higher to the mandible and the actuarial 5‐year mandibular necrosis rate in this group was 27%, albeit with a wide CI (95% CI, 2% to 52%). No cases of radiotherapy‐induced myelopathy were observed.

In univariate analysis, the following variables were associated with more late complications: a second primary malignancy as compared to a recurrence (40% vs 21%; *p* = .01), and the administration of concurrent chemotherapy versus not (60% vs 26%; *p* = .02). Remarkably, patients aged 65 years or younger at reirradiation experienced more complications (41% vs 2%; *p* = .02). Only 1 patient aged >65 years had a late complication.

### Event‐free survival

**Figure 4:**
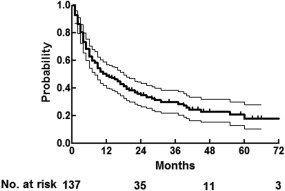
**Event‐free survival with 95% confidence interval**

Two‐year and 5‐year event‐free survival rates were 36% and 18%, respectively (Figure 4). This means that approximately one sixth of all patients survived at 5 years without tumor recurrence and without grade ≥4 late toxicity.

## DISCUSSION

This is one of the larger retrospective studies with a long‐term follow‐up investigating the efficacy of reirradiation in patients with a recurrent or a second primary head and neck malignancy. The importance of long‐term follow‐up for proper estimations of late complication and survival rates has been clearly documented.^16^, ^17^ Previously reported 2‐year locoregional control rates after reirradiation range from 10% to 64%, but long‐term follow‐up data are scarce.^11^, ^12^, ^18^, ^19^, ^20^, ^21^ OS rates in these reports vary from 10% to 58% depending on patient selection criteria. Severe late toxicity rates range from 8% to approximately 50%.^11^, ^12^ In the current cohort, favorable 2‐year and 5‐year locoregional control rates of 51% and 41%, respectively, were observed, with corresponding OS rates of 42% and 20%, respectively. The 5‐year actuarial serious late complication rate was 28%.

Currently, for previously irradiated patients with a disease recurrence, surgical resection is the treatment of choice. However, when patients present with surgically unresectable tumors, or if patients are unfit for surgery, reirradiation is the only available treatment with curative intent. Approximately one fourth of patients included in the current analysis were reirradiated without prior surgery, and 5‐year locoregional control and OS were only 20% and 14%, respectively. Poor outcome for this patient category was also reported by others.^18^, ^19^, ^22^ The dilemma in this situation is that adequate radiation dose for gross disease is often considered not safe and feasible because of the previous radiation treatment. Furthermore, the unfavorable selection based on advanced (unresectable) tumor and/or poor performance status predicts poor outcome. It should be noted that a favorable subselection in this group are patients reirradiated for a second primary tumor with a 5‐year locoregional control rate of 44%. In contrast, all patients primarily reirradiated for a recurring tumor incurred a locoregional recurrence, except for 1 patient with a follow‐up of only 7 months. This resulted in a locoregional control rate of 0% at 2 years. The likely explanation is that these recurrent tumors are a natural selection of the more aggressive and possibly also the more radioresistant types. It should be noted that this group of patients was relatively small (14 patients), no concurrent chemotherapy was administered, and patient selection may have played a strong role in the outcome. Others reported poor outcome for these patients as well, with 2‐year locoregional control rates of 14% and 19% with part of the patients receiving concurrent chemotherapy.^19^, ^23^ We emphasize that, if possible, surgery should be the treatment of choice for this category of patients.

The results of the subgroup of patients receiving postoperative reirradiation in addition to surgery are more favorable with 5‐year locoregional control and OS rates of 46% and 21%, respectively. These results are comparable with those in other studies.^23^, ^24^ Within this subgroup, patients with a recurrence undergoing a total laryngectomy experienced even better outcomes with a 5‐year locoregional control rate of 59%. Given these tumor control outcomes and acceptable toxicity rates, patients with high‐risk features, such as involved surgical margins or lymph node metastasis with extranodal growth, should definitely be considered for reirradiation.

The major limitations in head and neck reirradiation are disabling late toxicities. The actuarial incidence of grade ≥4 late toxicity in the current study was 28% at 5 years, which is within the range of 8% to 50% late toxicity previously described in literature.^11^ However, these late toxicity events are often presented as absolute rates and not as actuarial rates, which can be misleading. As the mortality in this group is high and patients will often be lost to follow‐up in their final disease stage, many patients are censored before late complications can occur. This might lead to a severe underestimation of the actual incidence of late toxicity when absolute rates are used. In literature, much higher 5‐year late complication rates ranging from 45% to 65% were reported when actuarial analysis methods were used.^22^, ^25^ It should be noted though, that for cohorts with relatively short survival, CIs of actuarial estimates increase with prolonged follow‐up because of decrease of the number of patients at risk.

ORN and mandibular ORN in particular, is one of the more frequently occurring late complications after reirradiation. Surprisingly, only limited data on mandibular ORN is available, and results are given in absolute rates only. De Crevoisier et al,^18^, ^24^ using conventional techniques, reported an absolute ORN rate of 8% to 16%. Salama et al^26^ reported an absolute mandibular ORN rate requiring surgery of 11% with a median cumulative radiation dose of 135 Gy in affected patients. However, no data on radiation exposure of the mandible were given. Another publication reported an absolute mandibular ORN rate of 4%, with a mean cumulative exposure of 109 Gy in affected patients.^20^ In the current cohort, no mandibular ORN was seen in patients receiving <100 Gy cumulative dose on the mandible. The 5‐year actuarial rate in patients receiving >100 Gy was 27%. This is significantly higher than the absolute ORN rates previously described, emphasizing the importance of the reporting of actuarial complication rates and selection criteria.

Three fatal treatment‐related complications were reported; 2 carotid blowouts and 1 bleeding because of soft tissue necrosis. Very high cumulative doses were administered at these complication sites (range, 128–130 Gy). No significant differences in complication rates were observed between patients with postoperative reirradiation and reirradiation alone. Approximately 1 of 4 surviving patients develops a severe late radiation toxicity (grades 4 and 5), which is comparable to rates reported in literature.^22^ Patients 65 years or younger incurred more complications in univariate analysis, which is the opposite of what is generally assumed. Radiation dose and follow‐up were equal in both groups, and no plausible explanation can be given for this observation.

Several studies have shown that a longer interval from the last radiotherapy treatment is correlated with an improved survival rate.^8^, ^18^ In the current study, in univariate analysis, an improved OS was observed if the time to reirradiation was >3 years (Table 2). No difference in locoregional control was observed. It should be noted that this group had a larger proportion of second primary tumors, which are known to be associated with an improved survival in comparison to tumor recurrences regardless of radiotherapy interval.^27^

In the current cohort, only a small proportion (5%) of patients received concurrent chemotherapy during reirradiation. In comparison, other retrospective reirradiation studies have reported higher rates ranging up to 80%.^21^ Although, in our center, every reirradiation patient is considered individually, therefore, the addition of concurrent chemotherapy for reirradiation is not considered standard of care, resulting in lower rates in comparison with other centers. For the limited amount of patients treated with chemotherapy in our cohort, no differences in tumor control or OS were observed. Univariate and multivariate analyses revealed chemotherapy to be associated with more late toxicity. Given the limited number of patients, this result should be interpreted with caution. Concurrent chemotherapy and reirradiation has been investigated in the phase II RTOG study 99‐11.^28^ Reirradiation with cisplatin and paclitaxel was given to 105 patients with recurrent or second primary head and neck cancers. The 2‐year OS rate was 26% and the 2‐year rate of grade ≥4 toxicity was around 30%. This is not clearly better than the results from the current study or other retrospective analyses.^29^, ^30^ In the postoperative reirradiation setting, Janot et al^31^ reported on 130 patients who underwent macroscopic radical salvage surgery, and were randomly assigned to either receive reirradiation combined with concomitant chemotherapy versus no adjuvant treatment. Locoregional control in the treatment arm was significantly better with increased toxicity, although no difference in OS was observed. In our cohort with postoperatively reirradiated patients with limited utilization of concurrent chemotherapy, locoregional control was comparable to this study. To our knowledge, there are no prospective studies directly comparing reirradiation alone with reirradiation plus concurrent chemotherapy. The role of chemotherapy and targeted therapies (eg, cetuximab), in the re‐treatment situation, thus remains an issue for further investigation.

The most common initial tumor site in our cohort was the larynx in more than half of the patients. In comparison to all other tumor sites taken together, locoregional control and OS rates were not significantly different (Table 2). Patients with other tumor sites were represented in smaller numbers, making analysis for these subgroups separately susceptible to bias. However, the patients with laryngeal carcinoma who underwent reirradiation after salvage laryngectomy did particularly well and this supports the notion that for patients with recurrent laryngeal carcinoma, laryngectomy is the first treatment option to be considered.

Forty‐four percent of the included patients were reirradiated with IMRT. As a result of improved normal tissue‐sparing using this technique, these patients could be treated with a higher dose at reirradiation. Although the cumulative radiation dose was higher, no increased rate of late complications was observed. Advanced radiation techniques, such as VMAT, proton, or particle therapy, may help to further reduce complications. In a recent in silico trial, a reduction in mean dose to organs at risk was achieved using particle therapy in the reirradiation setting.^32^ A borderline higher locoregional control was observed in patients treated with IMRT in univariate analysis, although this effect disappeared in multivariate analysis. A retrospective reirradiation series with 105 patients, however, did show an increased locoregional progression‐free survival in multivariate analysis for patients reirradiated with IMRT.^23^ A high‐precision radiotherapy technique, such as IMRT or VMAT should be used to optimize normal tissue‐sparing.

The results from the current study are subject to the inherent limitations of a retrospective analysis. The design resulted in a heterogeneous patient cohort with respect to histology, tumor site, tumor stage, and treatment. Patients who were primarily reirradiated were an unfavorable subgroup because they either had advanced irresectable disease or they were unfit for surgery. In the postoperative group, the surgery between 2 radiation treatments will have introduced uncertainties in dose calculation because irradiated tissues have been removed and healthy tissues may have been brought in for reconstruction. Further, the retrospective evaluation of late toxicity is difficult as there is always the risk of missing undocumented complications. However, missing severe late toxicity (grades 4 and 5) is unlikely, as these complications require medical care and are extensively documented in medical charts.

Another important limitation of this study was the incompleteness of dose‐volume data. This is a weakness of most other retrospective studies because to collect large enough cohorts with sufficient follow‐up, one needs to retrieve data well before the 3D era. There is no dispute that dose‐volume data are essential for further improvement of re‐treatment strategies.^33^ It is expected that with the general adoption of IMRT and better radiotherapy plan storage capacity, these data will become available soon.

The time‐range in which patients were reirradiated was 27 years, and, in this period, diagnostic and treatment protocols have changed. As knowledge on reirradiation has improved and it became more accepted during this timeframe, this will have had an effect on patients' and physicians' decision‐making regarding reirradiation resulting in a shift in patient selection criteria.

The strengths of this study are the long follow‐up and the large patient cohort.

It can be concluded that reirradiation should definitely be considered for patients with a recurrent or second primary head and neck cancer. Surgery is the treatment of choice for these patients with adjuvant reirradiation in case of high‐risk pathologic features. For patients who are not candidates for surgery, reirradiation alone is an option, albeit with less good prospects. This should be discussed with the patient balancing potential survival gain against the burden of treatment and the risk of complications.
